# Parallel neural pathways in higher visual centers of the *Drosophila* brain that mediate wavelength-specific behavior

**DOI:** 10.3389/fncir.2014.00008

**Published:** 2014-02-10

**Authors:** Hideo Otsuna, Kazunori Shinomiya, Kei Ito

**Affiliations:** ^1^Institute of Molecular and Cellular Biosciences (IMCB), University of TokyoTokyo, Japan; ^2^Department of Neurobiology and Anatomy, University of UtahSalt Lake City, UT, USA; ^3^Department of Psychology and Neuroscience, Life Sciences Centre, Dalhousie UniversityHalifax, NS, Canada

**Keywords:** Drosophila, phototaxis, wavelength-dependent, color vision, higher visual center

## Abstract

Compared with connections between the retinae and primary visual centers, relatively less is known in both mammals and insects about the functional segregation of neural pathways connecting primary and higher centers of the visual processing cascade. Here, using the *Drosophila* visual system as a model, we demonstrate two levels of parallel computation in the pathways that connect primary visual centers of the optic lobe to computational circuits embedded within deeper centers in the central brain. We show that a seemingly simple achromatic behavior, namely phototaxis, is under the control of several independent pathways, each of which is responsible for navigation towards unique wavelengths. Silencing just one pathway is enough to disturb phototaxis towards one characteristic monochromatic source, whereas phototactic behavior towards white light is not affected. The response spectrum of each demonstrable pathway is different from that of individual photoreceptors, suggesting subtractive computations. A choice assay between two colors showed that these pathways are responsible for navigation towards, but not for the detection itself of, the monochromatic light. The present study provides novel insights about how visual information is separated and processed in parallel to achieve robust control of an innate behavior.

## Introduction

**Figure 1:**
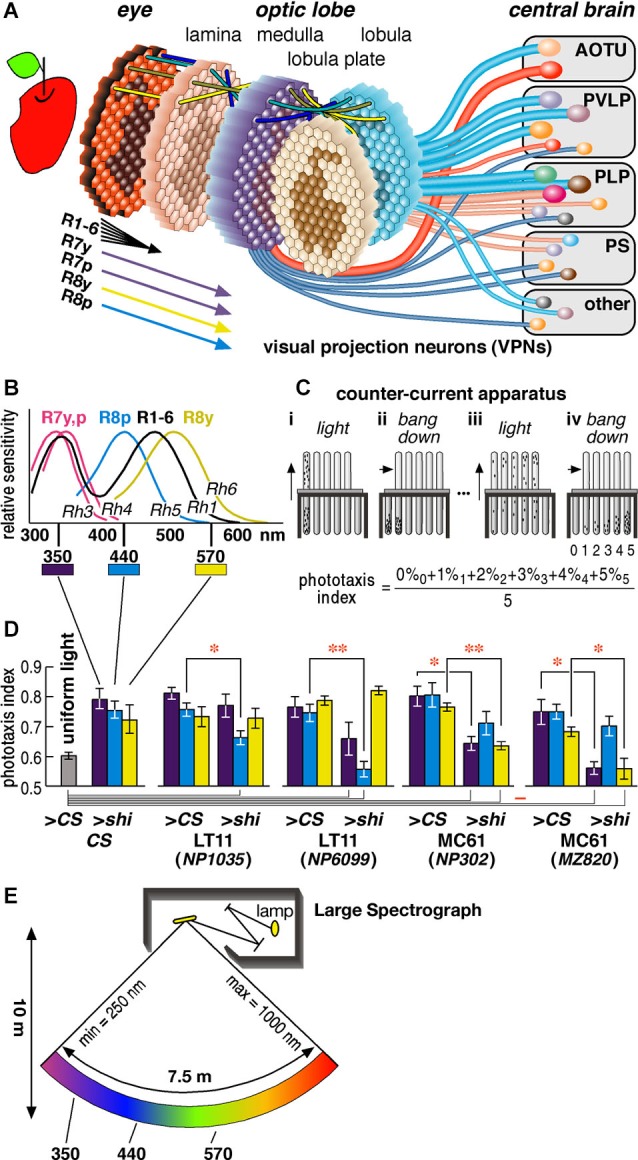
***Drosophila* visual system and phototaxis assay. (A)** Schematic diagram of the *Drosophila* visual system. Photoreceptors project from about 750 ommatidia of the retina to as many retinotopic columns (visual cartridges) in the four nested neuropils of the optic lobe. Relay interneurons project each retinotopic level onto the next (indicated by four colored lines on top of each neuropil). Hexagons with the projected images of an apple schematize the distribution of retinotopic visual cartridges and the relayed visual field. Cylindrical lines represent the visual projection neurons (VPNs) that connect the lower visual centers in the optic lobe to higher visual centers in the central brain (Otsuna and Ito, 2006). They terminate in the anterior optic tubercle (AOTU), posterior ventrolateral protocerebrum (PVLP), posterior lateral protocerebrum (PLP), posterior slope (PS) and a few other neuropils. The diameters of the lines reflect the numbers of neurons per pathway. Two red lines represent the pathways we analyzed in this study. **(B)** Sensitivity spectra of the photoreceptors**** (Salcedo et al., 1999). **(C)** The counter-current apparatus. Flies were put in the first tube and allowed to run towards the light for 30 s (panel i). The flies moved to the opposite tube were transferred to the bottom of the next tubes by tapping (ii) and let run towards light again (iii). After repeating this process five times (iv), flies were distributed to six tubes according to the times they moved towards light (0–5). **(D)** Phototaxis indices of the wild-type *CS* flies in the apparatus lit completely uniformly with white lamp (as negative control) and of the *CS* flies and GAL4 driver lines crossed with either *CS* (>*CS*, as positive control) or UAS-*shi^ts1^* (>*shi*) towards specific wavelengths of light (at 30^°^C). Mean ± SEM of three independent measurements with different sets of flies were shown. Statistical significance of differences by *t*-test is indicated with * (*p* < 0.05) and ** (*p* < 0.01) below the abscissa indicates the cases that were not significantly different from the behavior under uniform light (*p* > 0.05). **(E)** Schematic diagram of the Okazaki Large Spectrograph (OLS).

In animals ranging from insects to mammals, visual information is processed in a highly parallel manner. In certain mammals, segregated retinotopic pathways convey motion and color/contrast signals separately from the eye to the primary visual cortex (Zeki et al., 1991; Van Essen and Gallant, 1994), from where information is sent to multiple higher visual centers distributed in the occipital, parietal and temporal lobes that process different aspects of the information (Zeki et al., 1991; Born, 2001; Nassi and Callaway, 2006). Similarly, in insects, projections from the compound eye to nested neuropils of the optic lobe show clear retinotopic patterns, with each columnar visual cartridge in the optic lobe neuropils having specific projection from the retinal cells oriented to a specific angle of view (Figure 1A). These retinotopic projections involve many different morphologically and functionally distinct neurons (Fischbach and Dittrich, 1989; Otsuna and Ito, 2006; Strausfeld et al., 2006; Strausfeld and Okamura, 2007), which encode specific visual information such as figure-ground discrimination, motion detection, spectral information, and stereopsis (Krapp and Hengstenberg, 1996; Wicklein and Strausfeld, 2000; Douglass and Strausfeld, 2003; Strausfeld et al., 2006).

A compound eye of *Drosophila melanogaster* has about 750 ommatidia (*ca*. 730 and 780 in male and female eyes, respectively) (Wolff and Ready, 1993). Each ommatidium consists of eight photoreceptor cells that are called R1-R8. Axons of the R1-R6 cells from neighboring ommatidia, which receive light from the same direction of the visual field, converge into a single visual cartridge in the lamina. Axons of the R7 and R8 cells pass through the lamina cartridge and terminate in specific layers of the corresponding cartridge in the medulla. Relay interneurons connect the cartridges of different neuropils to mediate retinotopic signal transmission (Figure 1A).

The R1-R6 cells are essentially identical in that they express Rhodopsin 1 (Rh1) and have relatively broad spectral sensitivity (Salcedo et al., 1999) (Figure 1B). The R7 and R8 cells have two subtypes each that are called pale (p type) and yellow (y type) (Chou et al., 1996; Papatsenko et al., 1997; Chou et al., 1999). R7p and R7y cells express Rh3 or Rh4, respectively, and have sensitivity peaks at slightly different wavelengths in the ultraviolet (UV) range. R8p and R8y cells express blue-absorbing Rh5 and green-absorbing Rh6. The UV-, blue-, and green-sensitive R7 and R8 cells are regarded to play important roles in fly color vision (Bausenwein et al., 1992; Anderson and Laughlin, 2000; Strausfeld et al., 2006; Morante and Desplan, 2008; Yamaguchi et al., 2008, 2010), although R1-6 may in part be involved in color discrimination and R7/R8 may also play roles in motion discrimination (Wardill et al., 2012; Zhou et al., 2012).

Compared with connections between the retinae and primary visual centers, relatively less is known in both mammals and insects about the functional segregation of neural pathways connecting primary and higher centers of the visual processing cascade. In the insect nervous system, visual projection neurons (VPNs) connect the lower visual centers in the optic lobe to higher visual centers in the central brain (Figure 1A). A minimum of 44 types of VPNs, among which at least 20 are efferent, have been identified (Otsuna and Ito, 2006). Efferent VPNs terminate in the distributed but discrete centers in the central brain. These centers are called the optic glomeruli (Otsuna and Ito, 2006; Strausfeld and Okamura, 2007). Unlike optic lobe neuropils, each optic glomerulus does not have clear columnar arrangement. At this level of the visual system, therefore, simple retinotopic information is transformed to provide higher-level reconstructions about the visual world as in the connections between primary and higher visual cortices of the mammalian brain.

The relationship between the organization of identified neurons within a defined circuit and visual behaviors requiring specific nerve cells has been shown in the primary visual neuropils in the optic lobes (Rister et al., 2007; Yamaguchi et al., 2008; Zhou et al., 2012) and a higher integrative center, the brain’s central complex (Liu et al., 2006). Almost nothing, however, is known about the functional roles of neurons connecting these primary and integrative centers except for those mediating information about visual motion (Borst and Haag, 2002). What other functions are supported by these many efferent pathways? To address this question, we examined the roles of specific projection neurons in phototaxis, which is a robust innate behavior. Unlike random-walk phototaxis of unicellular organisms (Häder and Häder, 1988), flies determine the direction of the light source and walk straight towards it (Hotta and Benzer, 1970). Our analysis revealed that visual information required for proper phototaxis is mediated by multiple parallel pathways in a wavelength-specific manner and that phototactic responses towards ambient light and distant light source are handled differently.

## Materials and methods

### Fly stocks

GAL4 enhancer-trap strains with preferential expression in the efferent VPNs (Otsuna and Ito, 2006) were crossed with wild-type *Canton S* (*CS*) or a strain carrying upstream activation sequence (UAS)-*shibire^ts1^* (Kitamoto, 2001). Flies 4–8 days after eclosion were used for the behavioral assay.

To avoid the effect of UAS-*shibire^ts1^* during development, flies were reared on standard cornmeal-yeast-agar medium at 19^°^C (i.e., permissive temperature for *shibire^ts1^*) under 12/12 h light/dark intervals. Adult flies were collected within 1 day of eclosion and stored in batches of 45–50 flies per vial at 19^°^C. They were dark-adapted 1–2 h prior to behavioral assay. To avoid the effects of fatigue and possible learning behavior, flies were subjected to the phototaxis assay only once.

### Wavelength-specific phototaxis assay

**Figure 2:**
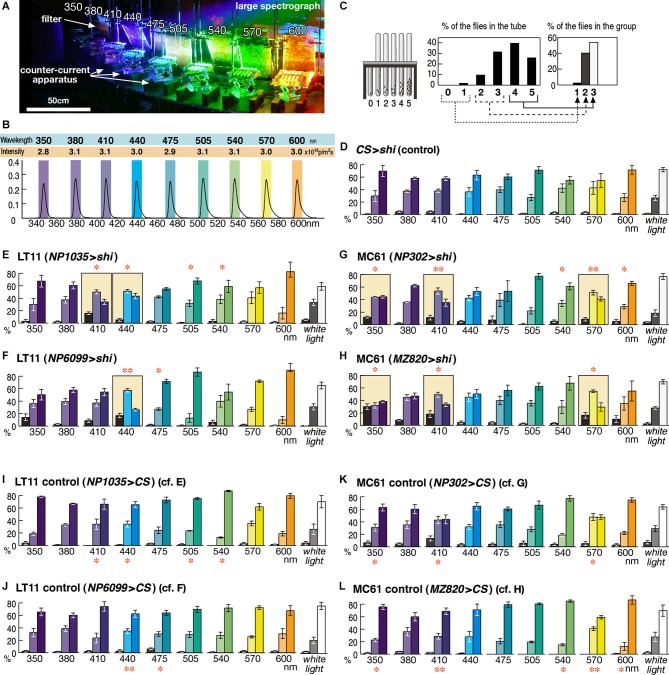
**Detailed phototaxis assay across the visual spectrum of the fly eye.** **(A)** Experimental setup of the OLS. Filters are placed in front of the counter-current apparatus to normalize the light intensity. For actual measurement, illumination except for a single apparatus was blocked in order to avoid the effect of stray light. **(B)** Intensity and spectrum of the light used for each experiment, measured at the center of the apparatus. Colored boxes indicate the wavelength range that illuminates each apparatus. **(C)** To minimize minor fluctuation of data, counts of the six tubes (0–5) are merged into three groups, which correspond to the flies that seldom moved towards light (left column, 1), moved or stayed roughly at random (middle, 2), and moved most of the time (right, 3). **(D)** Control experiment of the wild-type *CS* flies crossed with UAS-*shi^ts1^* (*CS* > *shi*) at 30^°^C. Mean ± SEM of three independent measurements. **(E–H)** Phenotypes of the GAL4 driver strains crossed with UAS-*shi^ts1^* at 30^°^C. Colored background rectangles indicate the cases in which flies showed aberrant phototaxis. The cases that were significantly different from the control **(I–L)** are indicated with ** (*p* < 0.01) and * (*p* < 0.05, *t*-test). **(I–L)** Control phototaxis experiment of **(E–H)**; each GAL4 line was crossed with wild-type *CS*. Phototaxis at 30^°^C was normal in all the cases.

The Okazaki Large Spectrograph facility (Watanabe et al., 1983) provides evenly dispersed monochromatic light between 250 and 1000 nm (Figures 1E, 2A) with wavelength specificity superior to regular band-pass filters and light-emitting diodes (Figure 2B). Counter-current analysis was performed using the original apparatus reported by Benzer (Benzer, 1967; Hotta and Benzer, 1970) (Figure 1C). We used polystyrene tubes (Falcon Round-Bottom tube 35-2917, ϕ17 × 100 mm) for visible range of light, and borosilicate tubes (Iwaki PYREX grass TE-32, ϕ16.5 × 105 mm) for ultraviolet light (350 and 380 nm) to avoid autofluorescence of the tubes. The tubes were cleaned thoroughly after each test with an ultrasonic washer in order to remove any remnants of the flies and their secretions.

The wavelength of light was calibrated with digital spectroradiometers USR-40V and USR-40D (Ushio). Because of the width of the counter-current apparatus, the wavelength peaks of the light for the first and sixth tubes were shifted by ±4 nm from that of the mid point (shown as colored rectangles in Figure 2B). Light intensity was measured with a digital photometer RMS-101 (Rayon) and adjusted to an equal level by placing neutral-density UV-transparent acrylic filters (Mitsubishi Rayon N083-085, 097, and 099, with 30–80% transmission) in front of the counter-current apparatus (Figure 2A). Because flies do not show clear phototaxis if the light is too strong or too weak, we adjusted the light intensity to the same level (3.0 × 10^18^ photons/m^2^s, Figure 2B) where wild-type flies showed clear phototaxis at all the wavelength ranges examined (Figure 2D). Phototaxis towards white light was tested using a 15 w fluorescent lamp driven by a 20,000 Hz inverter and placed 15 cm from the apparatus.

For the negative control experiment under uniform light (Figure 1D leftmost column), each tube was covered completely with semi-transparent paper, and the entire apparatus was lit from below and above with a pair of large light boxes.

Experiments with both *CS* and UAS-*shibire^ts1^* flies were performed at 30^°^C, i.e., the restrictive temperature of *shibire^ts1^*. A group of 45–50 flies were subjected to phototaxis assay at one time, and measurements with three independent sets of flies were averaged. All the strains showed normal anti-geotaxis behavior measured by the counter-current apparatus oriented vertically (data not shown).

### Selection of GAL4 driver lines

From the collection of 3,939 NP- and MZ-series GAL4 enhancer-trap strains, we first identified 96 lines that label specific subsets of VPNs but no other neurons in the retina or the optic lobe (Otsuna and Ito, 2006). We chose the strains for phototaxis assay with the following criteria: (1) Efferent pathways, which should convey visual information from the optic lobe to the brain, are labeled; (2) Only a single or maximum two such pathways are labeled; and (3) more than one such strain is available for labeling a particular pathway. We identified 15 such strains for analyzing six efferent pathways, and subjected them to the initial phototaxis experiments towards three wavelengths of monochromatic light (350, 440 and 570 nm). Pathways for which only a subset of the tested strains showed aberrant phenotype were excluded from further analysis.

### Visualization of GAL4-expressing cells

Confocal serial optical sections of whole-mount brains of the GAL4 strains expressing cytoplasmic UAS-green fluorescence protein (GFP) (S65T) or the combination of synaptic vesicle-targeted UAS-*n-Syb-GFP* and cytoplasmic UAS-*DsRed* were taken with a laser scanning microscope LSM510 (Zeiss) as described before (Otsuna and Ito, 2006). Neuropils were labeled with anti-Bruchpilot monoclonal antibody nc82 (Wagh et al., 2006). Single cells of the MC61 pathway were visualized using the flippase (FLP)-out system (Wong et al., 2002). Obtained serial section datasets were reconstructed using Imaris 2.7 (Bitplane) running on a Silicon Graphics Octane workstation or Fluorender 2.13 (Scientific Computing and Imaging Institute, University of Utah) running on a Windows PC. Definitions of neuropils were according to the systematic nomenclature of the insect brain proposed by the Insect Brain Name Working Group (Ito et al., 2014).

### Statistical analyses

**Figure 3:**
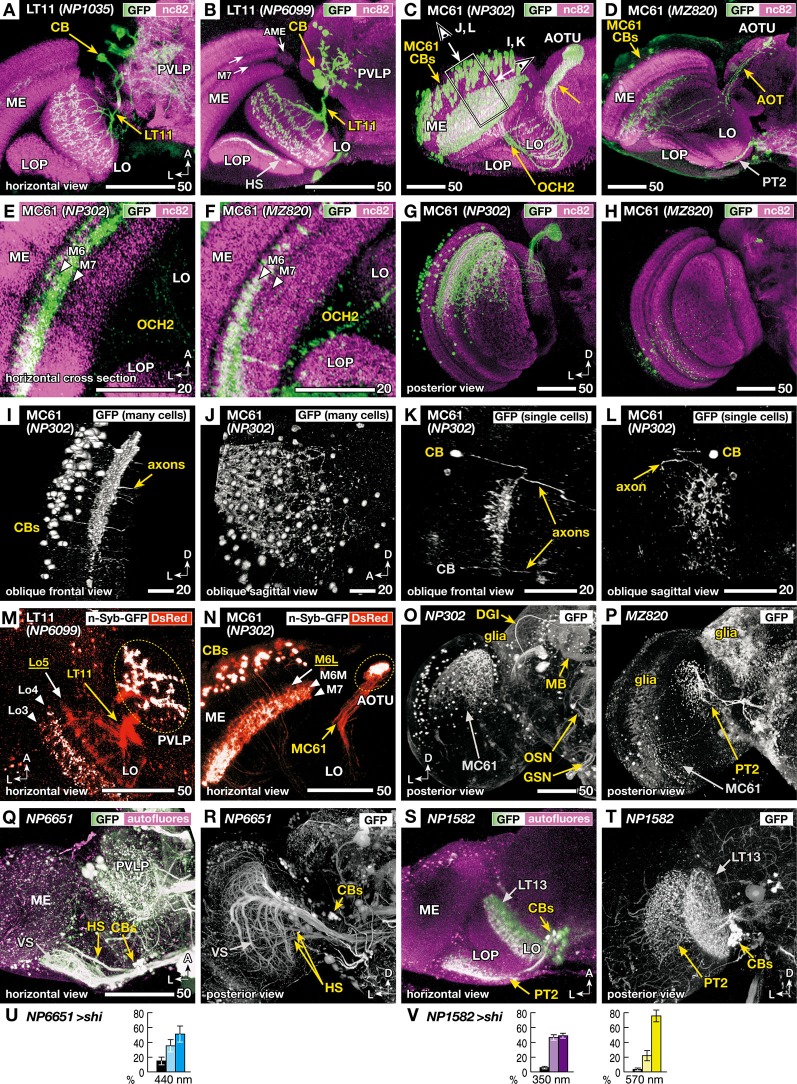
**Morphology of the VPNs labeled by the GAL4 lines with aberrant phototaxis**. Right-angled arrows on the bottom corners of the panels indicate directions in the images, A: anterior, L: lateral, D: dorsal. Scale bars correspond to either 20 or 50 μm. **(A–D)** Three-dimensional (3D) reconstruction of confocal laser scanning sections (horizontal view). Labeled neurons (green-white, visualized with GAL4-driven UAS-*GFP*) on the background labeling of synaptic neuropils (magenta, with anti-Bruchpilot nc82 antibody). CB indicates labeled cell bodies. In addition to the lobula tangential 11 (LT11) and medulla columnar 61 (MC61) VPNs, NP6099 and MZ820 strains labeled horizontal system (HS) neurons and lobula plate tangential 2 (PT2) neurons, respectively, in the optic lobe. Two arrows in **B** indicate dark-labeled layers in the background neuropil labeling of the medulla. Box and arrows in **C** indicate the region and the direction of oblique frontal views shown in Figures 3I, L. ME: medulla, AME: accessory medulla, LO: lobula, LOP: lobula plate, PVLP: posterior ventrolateral protocerebrum, AOT: anterior optic tract, AOTU: anterior optic tubercle, OCH2: second optic chiasmus. **(E, F)** High-magnification view of the horizontal sections of NP302 and MZ820 strains, showing specific arborizations in the medulla M6 and M7 layers (triangles). Note that the dendrites in the 3D reconstruction images of Figures 3C, D, G, H appear to extend more medially, because they visualize all the arborizations along the curved surface of the M6 and M7 layers. **(G, H)** Posterior 3D reconstruction of these strains, showing the labeling in different subsets of the MC61 neurons in the dorsal and ventral halves of the medulla. **(I, L)** 3D reconstruction views of the region shown as a box and arrows in Figure 3C; viewed along the axis that is parallel **(I, K)** or perpendicular **(J, L)** to the tangential plane of medulla layers. Entire population of MC61 neurons **(I, J)** and a sample with two FLP-induced (Wong et al., 2002) single-cell clones **(K, L)** are shown. In the latter, one of the cell bodies is out of the region of reconstruction (indicated with gray characters). **(M, N)** Distribution of presynaptic sites (white, visualized with synaptic vesicle-targeting UAS-*n-Syb-GFP*) and all the neural fibers (red, with cytoplasmic UAS-*DsRed*). Stacks of confocal horizontal sections. Lo3-5 and M6L, 6M and 7 indicate the layers of dendrites with (white) and without (yellow) presynaptic cites. Note that some of the cell bodies (CBs) are labeled with surplus amount of n-Syb-GFP. **(O–P)** Labeled cells in the central brain. In addition to the MC61 neurons, NP302 labels glial cells on the surface, dorsal giant interneuron (DGI), mushroom body neurons (MB), terminals of the olfactory sensory neurons (OSN) in the antennal lobe, and putative gustatory and other sensory neurons (GSN) in the gnathal (subesophageal) ganglia. MZ820 labels PT2 neurons as well as extensive glial cells on the surface. See Otsuna and Ito (2006) for the labeling pattern of NP1035 and NP6099. **(Q–V)** Labeling patterns and phototaxis phenotypes of other GAL4 driver strains that label HS and PT2 neurons, which are labeled simultaneously in the NP6099 and MZ820 strains, respectively, but not neurons of the LT11 or MC61 pathway. **Q, R** GAL4 strain NP6651 drives expression in the horizontal system cells (HS). The strain also labels the vertical system cells (VS; Note that in total 8–9 VS cells are observed, not 5–7 cells as previously described Heisenberg et al., 1978). **S, T** GAL4 strain NP1582 drives expression in PT2 neurons. LT13 neurons are also labeled. Reconstruction (horizontal view) of the labeled neurons (green-white, visualized with GAL4-driven UAS-*GFP*) on the background labeling of autofluorescence (magenta) **(Q, S)**, and posterior views without background labeling **(R, T)**. **(U, V)** Phototaxis behavior of NP6651 **(U)** towards 440 nm light and that of NP1582 towards 350- and 570-nm light **(V)**.

**Figure 4:**
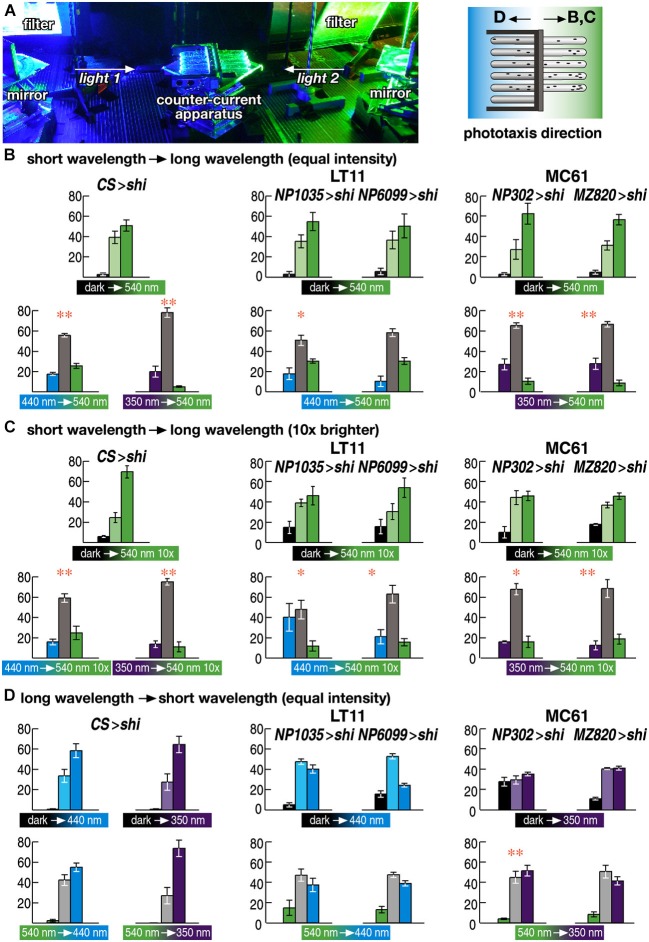
**Wavelength choice assay**. **(A)** Experimental setup. Two wavelengths of light are redirected using a pair of mirrors and introduced to the counter-current apparatus from both sides. Flies were allowed to run either from shorter to longer wavelength of light **(B, C)** or from longer to shorter wavelength **(D)**. **(B–D)** Choice assay between the wavelengths to which flies showed normal (540 nm) or aberrant phototaxis (440 and 350 nm for LT11 and MC61, respectively). Light intensity was adjusted to the same level as in Figure 3 (3 × 10^18^ p/m^2^s) in **(B, D)** but was set to 10 times brighter (3 × 10^19^ p/m^2^s) in **(D)**. Mean ± SEM of three independent measurements. Statistical significance of the differences between phototaxis (above) and choice assay (below) are indicated with ** (*p* < 0.01), * (*p* < 0.05; *t*-test).

Statistical significance of the differences between control and experiment (Figures 1, 2) and between phototaxis and wavelength choice assay (Figure 4) were examined using two-tailed *t*-test of the phototaxis indices. We assumed equal variance because of the results of *f*-test. Note, however, that *t*-test sometimes reported significance when both control and experiment data showed clear positive phototaxis but there were certain differences in the distribution of the flies (e.g., 505 and 540 nm of Figure 3E, 475 nm of Figure 3F, and 540 and 600 nm of Figure 3H).

For the phototaxis assay across fly’s visual spectrum, we performed 10 experiments for each experimental group (i.e., 350–600 nm and white light; Figure 2). Analysis of variance (ANOVA) of these 10 data sets showed no significance (*P* > 0.05) in the control groups (Figures 2D, I–L) but clear significance (*P* < 0.008) in all the experimental groups with *shibire* expression (Figures 2E, H).

When the counter-current analysis was performed under completely uniform lighting, the phototaxis index was higher than 0.5 (leftmost column of Figure 1D), as the distribution of the flies was shifted slightly towards the tubes that were further from the initial tube. This is likely because some flies try to escape from the area of the tube where they experienced shock by the tapping of the apparatus. Taking this effect into account, we determined aberrant phototaxis using the following criteria: (1) phototaxis index is not significantly different from that under uniform lighting (*P* > 0.05, *t*-test); (2) the index is significantly different from that of the control experiment with the GAL4 strains crossed with *CS* (*P* < 0.05, *t*-test); and (3) the percentage of the flies in the right column of the three-bar graph (i.e., the flies that moved towards light for four or five times out of five trials) is lower than in the middle column (i.e., those that moved two or three times out of five trials), or the right and left columns are about the same level and more than 10% of the flies remain in the left column (i.e., the flies that did not move or moved only once out of five trials). For example, phototaxis of the NP6099 > *shi* flies towards 350 nm light was considered normal even though the index was not significantly different from negative control (Figure 1D), because the index was not significantly different from positive control (NP6099 > *CS*), either (Figure 2J).

## Results

### Identification of neuronal pathways that are associated with phototaxis

Phototaxis can be assayed using the counter-current apparatus (Figure 1C) (Benzer, 1967). In this paradigm, flies in a tube are startled, or agitated, by tapping the apparatus, and they are left horizontally for certain period (typically 30 s) to stay in the same end or to run towards the other end of the tube that is facing the light. Flies that moved to the other end are transferred to the next tube, and the same tests are repeated. After five repetitive tests, most wild-type flies end up in the fourth or fifth tubes, showing that they chose to run towards light in 80% or 100% of the trials. The phototaxis index is calculated as the weighted average of the percentage of the flies in each tube. For wild-type flies, the index is above 0.7 if one end of the apparatus is lit by light, and 0.5–0.6 if the apparatus is lit evenly with uniform light (Figure 1D leftmost panel).

Mutant flies that show aberrant phototaxis have been screened using such apparatuses, and important mutants that affect development and fate determination of the retinal cells have been identified (Schümperli, 1973; Seiger and Woodruff, 1987; Ballinger and Benzer, 1988). However, such screening was unable to identify mutants that showed defects in the function or structure of the neural circuits deep within the brain. To identify neural pathways that are potentially associated with the phototaxis control, we therefore modified the assay in two respects.

First, instead of looking for gene mutations that are associated with phototaxis, we analyzed the roles of specific subsets of neurons by selectively silencing their functions. We expressed temperature-sensitive synaptic transmission inhibitor UAS-*shibire^ts1^* (*shi^ts1^*) (Kitamoto, 2001) with a series of GAL4 enhancer-trap driver strains that label certain VPNs but not retinal cells or intrinsic neurons of the optic lobe (Otsuna and Ito, 2006).

And second, because each ommatidium of the fly compound eye possesses photoreceptor cells with different wavelength sensitivity (Figure 1B; Salcedo et al., 1999), we suspected that phototaxis might be controlled in a color-dependent manner via separate neural pathways. We therefore tested phototaxis towards characteristic wavelengths of light that coincide with the sensitivity peak of photopic receptors: UV (350 nm), which correspond to the sensitivity peak (λmax) of the R7y/p photoreceptor cells, blue (440 nm) that is the λmax of R8p, and green (570 nm) that is detected predominantly by the R8y photoreceptors (Feiler et al., 1992; Salcedo et al., 1999).

To minimize the detection of light by other photopic receptors, the wavelength range of the light should be kept narrow. It is difficult to obtain such light with band-pass filters or light emitting diode (LED), which tends to have rather broad half bandwidth. Although a monochromator is ideally suited for this purpose, an ordinary one cannot provide monochromatic light for a broad enough area that covers the size of the counter current apparatus. To overcome this problem, we used the Okazaki Large Spectrograph (OLS) facility (Watanabe et al., 1983), which consists of a 30 kw xenon arc lamp and large interference grating and generates a spectrum from ultraviolet to infrared onto a platform that spans across 7.5 m (Figure 1E). By placing the counter current apparatus onto the platform, it is possible to perform phototaxis assay towards specific wavelength of light (Figures 2A, B).

Using the OLS, we identified two pairs of GAL4 driver strains that drove expression in the same visual pathways and showed identical phenotypes (Figure 1D). Two strains (NP1035 and NP6099) with GAL4 expression in a specific VPN pathway called Lobula Tangential 11 neurons (LT11) showed aberrant phototaxis (i.e., low phototaxis index) towards blue light (440 nm) when crossed with the UAS-*shibire^ts1^* strain (Figure 1D middle panels). The observed phenotype should not be due to general locomotion defects, because the phototaxis of these flies towards UV or green light (350 and 570 nm) was not affected significantly. The other two lines (NP302 and MZ820) had preferential expression in a newly identified neuron type that we named Medulla Columnar 61 neurons (MC61). They showed aberrant phototaxis towards UV and green light, but not towards blue light (Figure 1D right panels).

### Spectral responses of the phototaxis-associated visual projection neurons (VPNs)

Neurons silenced by these GAL4 driver strains are likely to be necessary specifically for phototaxis towards particular wavelength ranges. As shown in Figure 1D, the responses of the LT11 and MC61 pathways appear complementary: LT11 for blue and MC61 for UV/green. Possibly, this suggests that the two pathways convey information about primary colors involving a form of color opponency mechanism (Fischbach, 1979). To explore this, we tested the response spectrum of these neurons at higher resolution by subjecting flies to the phototaxis assay at ∼30 nm intervals (Figure 2).

When the control flies that carry UAS-*shibire^ts1^* but without GAL4 driver were subjected to this assay, they showed normal phototaxis behavior not only towards white light but also towards monochromatic light across all the visible ranges from 350 to 600 nm (Figure 2D). Functional knockout of LT11 neurons caused aberrant phototaxis specifically at 410- and 440-nm ranges (for NP1035, Figure 2E) or at 440 nm (for NP6099, Figure 2F). Although these wavelengths correspond to the λmax of the R8p cells (Salcedo et al., 1999), the wavelength dependence appears to be more specific than the sensitivity spectrum of the R8p photoreceptor (Figure 1B).

The response to MC61 knockout was even more intriguing (Figures 2G, H). Though the MC61-silenced flies showed defective phototaxis at 350 nm, they showed a normal response at a slightly longer UV wavelength (380 nm). They again showed defects towards violet light (410 nm), but behaved normally towards blue (440 nm). Their phototaxis further showed defects in green light (570 nm), but behavior at slightly different wavelengths (470–540 nm and 600 nm) was normal. The characteristic wavelength dependency was identical between the two independent driver strains.

It is important to note that phototaxis towards mixed white light was never affected in all the four strains and that the flies showed normal phototaxis in many wavelength ranges. These indicate that the components of the nervous system that are responsible for locomotion control and for the phototaxis towards these colors of light are kept intact in these flies.

### Architecture of the phototaxis-associated visual projection neurons (VPNs)

We next examined the detailed architecture of the LT11 and MC61 pathways. As described earlier, the LT11 pathway consists of a single unique neuron (Otsuna and Ito, 2006), with a cell body in the lateral cell body rind, tree-like arborization in the lobula that spans across the entire relayed visual field, and axon terminals in the posterior ventrolateral protocerebrum (PVLP) of the central brain (Figures 3A, B).

The MC61 pathway has not been described before. It is significantly different from LT11 in that it consists not of one neuron but of an ensemble of many isomorphic neurons. They have their cell bodies in the lateral surface of the medulla. These neurons send their neurites in parallel with the medulla visual cartridges and form extensive dendritic arborizations in its middle layers (Figures 3C, D). Background labeling with nc82 antibody, which labels neuropils according to the density of the active zone protein Bruchpilot (Wagh et al., 2006), shows two bands that appear darker in the medulla (two arrows in Figure 3B). Because of its location that is closely associated with the accessory medulla (AME) at its anterior end, and the relative paucity of synapses because of the massive tangential axon fibers of the serpentine layer that run in this region, the medial band corresponds to the medulla M7 layer. The region just lateral to this layer is labeled densely because of the abundant presynaptic sites of the retinal R7 photoreceptors (Fischbach, 1979) and is called the medulla M6 layer. High-magnification cross section views of this region indicate that the dendrites of the MC61 neurons arborize in these two layers (Figures 3E, F).

Axons arising from different optic cartridges of the medulla converge at the second optic chiasmus (OCH2; Figures 3C, E, F) but are again get dispersed to pass through the lobula in a mutually parallel manner at various positions (Figures 3C, D). The axons again converge at the root of the anterior optic tract and terminate in the lateral zone of the anterior optic tubercle (AOTU).

Interestingly, the two strains label different subsets of isomorphic MC61 neurons (Figures 3G, H). NP302 strain labeled *ca*. 120 neurons (111, 120 and 125 cells observed in 3 samples) lying in the dorsal half of the medulla (Figure 3G), whereas MZ820 labeled *ca*. 13 neurons (10, 14 and 16 cells observed in three samples) that are scattered more sparsely in the ventral medulla half. Thus, the MC61 neurons labeled in the two strains preferentially process information from the dorsal and ventral halves of the visual field, respectively. In the AOTU, axons labeled in the NP302 strain are spread almost in its entire cross section, whereas those labeled in MZ820 are confined in its dorsal region.

To analyze the dendrites of MC61 neurons in relation to medulla layers, reconstructions viewed from the angles parallel and perpendicular to these layers were examined (Figures 3I–L). Because the tangential medulla layer spans obliquely from anterior-medial to posterior-lateral, oblique viewing angles were employed (arrows in Figure 3C). The columnar arrangement of the neurites of the MC61 neurons, as well as their dendrites spanning tangentially in specific layers, were apparent when the sample was observed in a view parallel to the layer (Figure 3I). When the same sample was viewed at a right angle to the layer, the distribution of the MC61 somata appeared disorganized (Figure 3J). Indeed, single-cell labeling showed that tangential dendrites of each neuron arborized in specific subregions of the relayed visual field (Figures 3K, L), which is slightly offset compared to the position of the somata. The extent of these dendrites covered about 1% of the tangential cross-section area of the medulla (0.8% and 1.15% in two samples), suggesting that they span roughly across eight out of the total *ca*. 750 medulla visual cartridges.

We then analyzed the distribution of putative input and output sites of these neurons by comparing the labeling patterns of cytoplasmic *GFP* and synaptic vesicle-targeted reporter *n-Syb-GFP* (Figures 3M, N). Because the transgenic n-Syb-GFP protein is produced in the cell body and transported to the distal synaptic sites, surplus molecules tend to visualize somata (Figure 3N). In spite of this, within the neurites the protein is distributed specifically to the presynaptic sites, while axons and postsynaptic sites-specific regions of dendrites are left unlabeled. The dendrites of LT11 reside in three discrete layers (Lo3, 4 and 5 layers) of the lobula (Figure 3M). Presynaptic sites in this neuron were observed not only in its terminals in the PVLP but also in two layers of the lobula (Lo3 and Lo4), suggesting that its distal branches in the lobula outer layers are both receiving and imparting information. Likewise, the MC61 neurons had presynaptic sites both in the distal target in the AOTU and in the proximal dendrites in the medulla (Figure 3N). Although MC61 neurons arborized in two layers of medulla (M6 and M7 layers, Figure 3E), presynaptic sites were confined only in the M7 layer and the medial half of the M6 layer (M6M; Figure 3N).

Thus, for both LT11 and MC61 pathways, one level of their arborizations in, respectively, the lobula Lo5 layer and the lateral half of the medulla M6 layer (M6L) were devoid of presynaptic sites and were therefore exclusively dendritic (Figures 3M, N). That efferent neurons also possess discrete levels of presynaptic processes within what looks like a dendritic tree indicates the participation of these neurons also in local computation of the visual signal within the primary visual centers.

As mentioned above, the four strains showed no GAL4 expression in the photoreceptors or intrinsic neurons of the optic lobe. However, some lines label other types of VPNs in addition to the LT11 and MC61: The NP6099 strain labels the horizontal system (HS) cells (Figure 3B), whereas MZ820 labels lobula plate tangential 2 (PT2) neurons (Figures 3D, P). To ask whether these additionally labeled neurons might affect the observed phenotype, we looked for other GAL4 driver strains that label these neurons. The NP6651 strain labels HS as well as the vertical system (VS) cells, but not the LT11 neuron (Figures 3Q, R). Unlike NP1035 and NP6099 strains, phototaxis towards 440 nm light was normal (Figure 3U), suggesting that the observed phenotype in NP6099 was not because of the silenced HS cells. Likewise, silencing PT2 as well as LT13 neurons with the NP1582 driver strain (Figures 3S, T) caused no aberrant phototaxis (Figure 3V).

The four driver lines also labeled a few other neurons or glial cells in the central brain. Because there was no overlap in these central brain neurons that were labeled between NP1035 and NP6099 strains (Otsuna and Ito, 2006) and between NP302 and MZ820 strains (Figures 3O, P), these cells were unlikely to be the cause of the observed phenotype that are common within each pair of strains. None of these strains labeled descending motor control neurons projecting from the brain to the thoracico-abdominal ganglia.

### Role of the phototaxis-associated visual projection neurons (VPNs) in color-choice test

Why, then, do silencing particular visual neural circuits cause phototactic defects at particular wavelengths? Do the flies become blind to these colors of light? Or, are they able to recognize the source of illumination but unable to navigate towards that direction? To distinguish which of the two is more likely, we performed a choice assay between two wavelengths of light (Figure 4).

Wild-type flies prefer shorter wavelength of light (Schümperli, 1973; Fischbach, 1979). Consistent with this, phototaxis of the wild-type flies from dark towards 540 nm green light was lost, if the flies were asked to run from the end of the counter-current apparatus that was lit by blue (440 nm) or UV (350 nm) light towards the end that was lit by green light (Figure 4B left panels).

Interestingly, flies with silenced LT11 or MC61 pathways showed the same phenotype (Figure 4B middle and right panels). When the flies were placed in the end of the apparatus that was lit by blue or UV light, respectively, and the other end was lit by green light, many flies remained in the blue or UV-lit end and did not run towards green light, even though their phototaxis behavior was normal towards green light but defect towards blue or UV (Figures 2E, H).

This apparently contradicting phenotype could be because these flies might have lower sensitivity towards green light than wild-type flies do, causing the misinterpretation that blue or UV light might appear brighter to them. To test whether this would be the case, we increased the intensity of green light tenfold. The phenotype remained essentially the same, however (Figure 4C). Thus, these flies were able to determine that blue or UV light is preferable to green light.

We next placed the flies in the end of the tubes that was lit by green light and asked the flies to run towards the end that was lit by blue or UV light. Even though they were able to stay in the end that was lit by shorter wavelengths of light, they failed to run from green-lit towards blue- or UV-lit ends (Figure 4D). These data indicate that only navigation towards shorter wavelength of light, but not the detection of ambient light, was impaired by silencing these neural circuits.

## Discussion

The current study identified parallel pathways of the visual neural circuits that mediate phototaxis towards highly specific wavelength ranges. The finding is intriguing in three respects.

First, we found that seemingly achromatic behavior such as phototaxis is actually mediated by multiple parallel pathways, each of which responds to only specific colors of light (Figure 2). The visual signals conveyed by these neurons are distinct from the spectral sensitivity curves of the photoreceptors. Their spectral responses are so narrowly tuned that animals living in the natural environment would seldom encounter with such monochromatic illumination. Thus, the two pathways should function most of the time in parallel. Such parallel computing is advantageous for the animals, because it should help achieving the robust control of innate behaviors; malfunction of any of these parallel pathways should not affect the fly’s response towards mixed light.

Secondly, the pathways we identified are responsible for the navigation towards the light source, but not the detection of the ambient light (Figure 4). Phototaxis behavior has been analyzed in two paradigms. In the so-called “slow” phototaxis, animals are put at the center of the tube or the branch point of the T- or Y-maze, and asked to move towards either direction (Fischbach, 1979). In this paradigm the choice of both ends involves navigation through the tubes. On the contrary, in the startled phototaxis such as the counter-current paradigm we used, flies are put in one end of the tube and asked either to stay there or to move towards the other end of the tube. Using the latter paradigm, we were able to distinguish the flies’ preference towards specific color of light between the situations when it is presented as an ambient light around where the flies are placed, or when it is presented in the other end of the tube as a distant target. Silencing of the two pathways caused defects only in the latter case. Detection of the colors of the ambient light might be mediated by other, yet unidentified, visual pathways.

The separation of visual processing between ambient color and target color is reminiscent of the motion detection, in which movement of the large background objects and small target objects are handled separately (Kirschfeld, 1994; Fox and Frye, 2013). The fact that the two pathways analyzed here are involved only in the detection of the target light source may infer that they might be involved in the distinction of the colors of distant objects. If this is the case, then the narrowly tuned wavelength responses should be useful for examining the reflection spectra of the target objects.

Thirdly, even though the two pathways seem to function in parallel, they are drastically different in terms of the visual information they convey. The LT11 pathway consists of a single neuron that extends dendrites across the entire relayed visual field. Given its structure, the single neuron cannot convey information about the distribution of light in the visual field (Otsuna and Ito, 2006). The MC61 pathway, on the contrary, consists of more than 100 columnar neurons. Though they are somewhat fewer than the number of the *ca*. 750 visual cartridges, the pathway might be able to transmit retinotopic information albeit at a lower resolution. Their terminals in the AOTU are rather intermingled. Although the terminals of the MC61 neurons arising from the ventral medulla, labeled in the MZ820 strain, are confined in the dorsal region of the AOTU suggesting some sort of retinotopic projection, the terminals labeled in the NP203 strain essentially cover the entire cross section of the AOTU even though these axons derive from only the dorsal half of the medulla. It is not yet clear whether precise retinotopic projection is maintained by these neurons.

The two pathways are contrasting also in their target neuropils. Whereas LT11 terminate in the PVLP, MC61 innervate the AOTU. The AOTU is one of the most prominent optic glomeruli, and bundles of neural fibers project from the AOTU towards the region called the bulb, or the lateral triangle, where they contact with the neurons of the ellipsoid body (Ito et al., 2013). Thus, information sent via the MC61 pathway is likely to be transmitted to the central complex, which is known to be important for higher-order visual processing (Liu et al., 2006; Heinze and Homberg, 2007). On the contrary, the PVLP has no direct connection with the bulb or the central complex, although it is connected with various other neuropils (Ito et al., 2013).

The observed phenotypes in terms of a lack of movement towards the light, as opposed to lack of detection *per se*, suggest potential involvement of the downstream neural circuits from visual to motor centers. Because the examined flies showed normal phototaxis towards white as well as towards many specific wavelength ranges of light, and because the four strains we used did not label overlapping neural circuits in the central brain, it is not likely that the downstream neural circuits in the potential higher visual or motor centers were affected in our assay. Further identification of the information pathways arising from the target regions of the LT11 and MZ61 neurons to higher visual/motor centers would be required to understand how wavelength-specific information is utilized for behavior control.

The parallel pathways also differ in the location of their input sites. Whereas LT11 arborizes in the Lo3, 4 and 5 layers of the lobula, MC61 neurons receive information in the M6 and M7 layers of the medulla. The sharp and complex response spectra of LT11 and MC61 neurons should require subtraction of signals deriving from different types of photoreceptors, as has been described for chromatic sensitive ganglion cells in higher mammals (Martin et al., 2001). Indeed, the layers in the medulla and lobula in which these neurons have dendrites receive inputs from neurons relaying information from R7 and R8 photoreceptors (Bausenwein et al., 1992; Gao et al., 2008; Shinomiya et al., 2011).

In spite of their structural differences, the two pathways share one common characteristic: neurons of both pathways can gather information from multiple visual cartridges. The single LT11 neuron arborizes across the entire visual cartridges of the lobula. Even though the MC61 neurons have columnar organization, a single neuron extends its dendrites tangentially across about eight neighboring visual cartridges (Figures 3K, L). Considering the existence of extensive pre- and post-synaptic sites in these dendrites (Figures 3M, N), it is likely that these dendrites should take part in complex computation to compare light intensity between different parts of relayed visual fields. Not only global comparison of the entire visual field by the LT11 neuron but also local comparison by each of the MC61 neurons will provide information about the gradient of light intensity, which is a decisive cue for determining the direction of light source for phototaxis.

In this study we used two driver strains for assessing the role of each pathway. The observed phenotypes were slightly different between drivers. Concerning the LT11 pathway, NP1035 strain caused defect phototaxis at two wavelength ranges (410 and 440 nm), whereas NP6099 showed defects only at the latter (Figures 2E, F). As for the MC61 pathway (Figures 2G, H), although the two strains showed aberrant phototaxis at the same sets of wavelengths, defects towards 350- and 570-nm light were slightly weaker with NP302 than with MZ820. (Rightmost bars in the triplet graphs at these wavelengths in Figure 2G were higher than the leftmost bar, indicating that slightly more flies ran towards light, whereas the right and left bars in Figure 2H had similar heights). Stronger phenotype with MZ820 is interesting, because it drives expression in much fewer number of MC61 neurons compared to NP302.

Recent connectomics analysis has provided highly comprehensive information about the connections of medulla-associated neurons (Takemura et al., 2013). Considering their dendritic arborization patterns, the MC61 neurons should be included in the volume of the electron microscope preparation analyzed in that study, although it is not possible at this state to identify them due to the lack of information about the projection targets outside of the analyzed volume.

Because of the technical difficulty, we were not able to examine the activity of LT11 and MC61 neurons in response to various wavelengths of light. AOTU of the honeybee brain was recently reported to show spectral responses (Mota et al., 2013). Technical sophistication in the future may reveal wavelength-dependent activity of LT11/MC61 neurons and their possible downstream partners.

Neural mechanisms underlying even a simple behavior like phototaxis have not been well understood. In spite of the recent advances in the understanding of the neural networks involved in color vision, little is yet known about how such information is conveyed to higher centers. Our study has provided a first glimpse into how multiple higher visual centers receive visual signals that encode complex responses to spectra in a complementary manner. Further analyses of the synaptic organization of neural circuits associated with the neurons described above will reveal how such computations are achieved and will shed broader insight into the principles of how the brain reconstructs the visual world.

## Conflict of interest statement

The authors declare that the research was conducted in the absence of any commercial or financial relationships that could be construed as a potential conflict of interest.
